# Real-world treatment outcomes for Hodgkin lymphoma in South Africa: a prospective observational study

**DOI:** 10.1186/s13027-024-00612-4

**Published:** 2024-09-27

**Authors:** Samantha L. Vogt, Garrick Laudin, Marianna Zahurak, Jenifer Vaughan, Atul Lakha, Sugeshnee Pather, Ziyaad Waja, Deshan Chetty, Tanvier Omar, Wendy Stevens, Philippa Ashmore, Kennedy Otwombe, Khuthadzo Hlongwane, Ravi Varadhan, Moosa Patel, Richard F. Ambinder, Neil A. Martinson, Rena R. Xian, Vinitha Philip

**Affiliations:** 1 Department of Medicine, Johns Hopkins School of Medicine, Division of Hematology, 4940 Eastern Ave, Rm 4500, Baltimore, MD 21224 USA; 2 Department of Oncology, Sidney Kimmel Comprehensive Cancer Center, Johns Hopkins School of Medicine, Baltimore, MD USA; 3 Perinatal HIV Research Unit (PHRU), Faculty of Health Sciences, University of the Witwatersrand, Johannesburg, South Africa; 4 Clinical Haematology Unit, Department of Medicine, Chris Hani Baragwanath Academic Hospital and Faculty of Health Sciences, University of the Witwatersrand, Johannesburg, South Africa; 5 Department of Molecular Medicine and Haematology, Faculty of Health Sciences, National Health Laboratory Service, University of the Witwatersrand, Johannesburg, South Africa; 6 Division of Anatomical Pathology, Faculty of Health Sciences, National Health Laboratory Service, University of the Witwatersrand, Johannesburg, South Africa; 7 Wits Diagnostic Innovation Hub, Faculty of Health Sciences, University of the Witwatersrand, Johannesburg, South Africa; 8 Clinical Haematology, Netcare Olivedale Hospital, Johannesburg, South Africa; 9 School of Public Health, Faculty of Health Sciences, University of the Witwatersrand, Johannesburg, South Africa; 10 Department of Pathology, Johns Hopkins School of Medicine, Baltimore, MD USA

**Keywords:** HIV, Hodgkin lymphoma, Tuberculosis, Bone marrow involvement, Overall survival

## Abstract

**Background:**

Prospective data from sub-Saharan Africa suggests that treatment outcomes for people living with HIV (PWH) with Hodgkin lymphoma (HL) are similar to those without HIV. However, real-world data from high-resource settings and retrospective studies from sub-Saharan Africa, suggest inferior outcomes. We set out to evaluate the real-world treatment outcomes for HL in South Africa to better understand the disparate outcomes.

**Methods:**

We established a prospective, observational cohort of newly diagnosed, adult (≥ 18 years) HL cases recruited from Chris Hani Baragwanath Academic and Netcare Olivedale Hospitals in Johannesburg, South Africa between March 2021 and March 2023. Participants were followed for up to 18 months after enrollment with data censored on December 23rd, 2023. The primary endpoint was 1-year overall survival.

**Results:**

We enrolled 47 participants with HL including 31 PWH and 16 HIV-negative. Advanced stage disease and B symptoms were common at time of diagnosis irrespective of HIV status. Bone marrow biopsy, performed during the work-up and evaluation of cytopenias, provided the initial diagnosis of HL in 16/31 (52%) PWH. HIV status and bone marrow involvement were associated with early mortality (within 3 months of diagnosis) and a poorer 1-year overall survival from diagnosis (HIV: 55% vs. 88%; *p* = 0.03; bone marrow involvement: 50% vs. 80%; *p* = 0.02). Among evaluable participants, those that received at least 6 cycles of chemotherapy and underwent response assessment, there was no difference between those with and without HIV.

**Conclusion:**

Traditional laboratory markers of poor prognosis including anemia, lymphopenia and hypoalbuminemia were more common among PWH and those with bone marrow involvement and suggest high risk disease. A better understanding of the drivers of these aggressive presentations is warranted to ensure more PWH are able to tolerate chemotherapy.

**Supplementary Information:**

The online version contains supplementary material available at 10.1186/s13027-024-00612-4.

## Background

HIV is associated with an increased incidence of Hodgkin lymphoma (HL) [1]. In high-resource settings, a decrease in non-Hodgkin lymphoma incidence was observed with the advent of antiretroviral therapy (ART) [2, 3]. In contrast, the incidence of HL has been stable to increased in the ART era [4, 5]. A similar trend for HL has been noted in South Africa since the widespread availability of ART in 2005 [6].

Advanced stage presentations, along with prior and current tuberculosis (TB) disease are more common in people with HIV (PWH) and HL in South Africa [7]. Retrospective studies suggest that approximately 19–38% of PWH with HL have active TB disease at the time of HL diagnosis compared to 6% of HIV-negative HL participants [7, 8]. The empiric treatment of TB in PWH with lymphadenopathy and constitutional symptoms has been proposed as a possible contributor to the advanced stage lymphoma presentations seen in the region [8].

Observational studies and clinical trial data from high-resource settings show similar outcomes for HL regardless of HIV status in the ART era [9, 10], while real-world data suggest a disparity in treatment outcomes [2, 11, 12]. Prospective data from Africa are limited but suggest similar outcomes regardless of HIV status [13, 14], while retrospective studies from South Africa have suggested poorer outcomes for PWH and HL [6–8]. To better understand these disparate outcomes, we report on the real-world treatment outcomes for newly diagnosed HL participants in Johannesburg, South Africa.

## Methods

### Study population

**Fig. 1:**
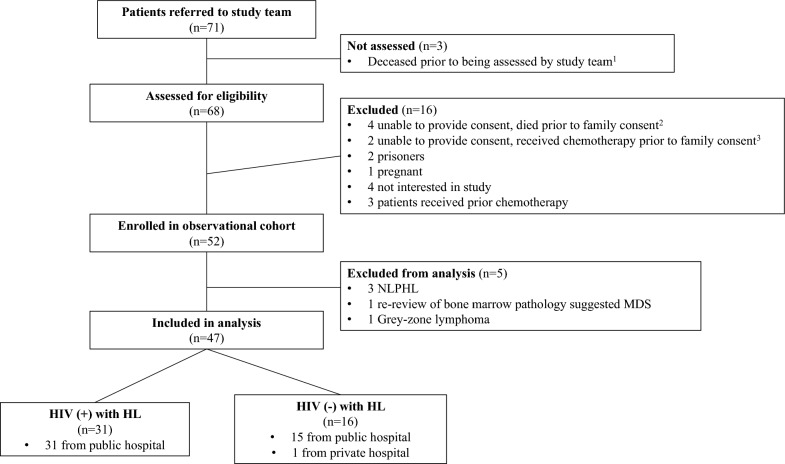
Referral and eligibility flow chart. *NLPHL* Nodular lymphocyte predominant Hodgkin lymphoma; *MDS* Myelodysplastic syndrome; *HL* Hodgkin lymphoma. ^1^All 3 cases were HIV-associated and noted to have bone marrow involvement. ^2^Three cases were HIV-associated and noted to have bone marrow involvement, 1 case was in an elderly HIV-negative individual. ^3^Both cases were HIV-associated and noted to have bone marrow involvement and disseminated tuberculosis

We conducted a prospective, observational study of newly diagnosed Hodgkin Lymphoma (HL) cases referred from the department of Haematology at Chris Hani Baragwanath Academic and Netcare Olivedale Hospitals in Johannesburg, South Africa. Chris Hani Baragwanath Academic Hospital (CHBAH) is a tertiary hospital in the public sector, while Netcare Olivedale Hospital is a private sector hospital. Between March 2021 and March 2023, 71 participants with pathologically confirmed HL were referred to the study team and 52 were enrolled. Following pathologic review, five participants were excluded from the analysis. Reasons for non-enrollment and exclusion are listed in Fig. 1.

Eligibility criteria included age ≥ 18 years. Family consent was permitted for participants unable to provide consent due to deteriorating clinical status. Consent forms were offered in English, Zulu, and Sestho. Exclusion criteria included an inability to provide consent (by participant or family member), incarceration, pregnancy or receipt of prior chemotherapy. This study was approved by the Johns Hopkins School of Medicine Institutional Review Board (IRB00107027) and the University of the Witwatersrand Human Research Ethics Committee (200208).

At enrollment, demographic information was collected and an Eastern Cooperative Oncology Group (ECOG) performance status (PS) assessment was performed. Demographics included sex assigned at birth and self-identified race. Consent for medical record review, including clinical notes, laboratory and radiology data, for up to 18 months after enrollment and access to archived tumor biopsy material was obtained. Laboratory review included HIV status, CD4 count and HIV viral load (if HIV positive), full blood count, and complete metabolic panel. Pathology reports were reviewed to confirm the diagnosis and confirm EBV status of the tumor. Standard pathologic confirmation of HL in South Africa includes immunohistochemistry (IHC) assessment of CD3, CD20, CD15, CD30 and PAX5 [15]. Staging work-up included bone marrow biopsy (BMB) and whole body imaging (CT or FDG-PET/CT) and followed the Lugano classification [16]. Cardiac ejection fraction (EF) was measured by transthoracic echocardiogram. The International Prognostic Score (IPS) [17] was calculated using the abstracted risk factors. TB diagnosis was defined as any of the following: GeneXpert result of positive or trace, positive Ziehl–Neelsen (ZN) stain for acid-fast bacilli or positive urine lipoarabinomannan (LAM). Treatment of TB is monitored through the National Institute for Communicable Disease and participants are assigned a local clinic for follow-up. Dosages of TB medicines are monitored by the local clinic. When complications of TB treatment are suspected, such as drug-induced liver injury or Isoniazid-mono resistance, these cases are referred to an Infectious Disease specialist for management.

### Specimen collection and processing

Participants provided an expectorated sputum sample for TB testing that included GeneXpert Ultra and mycobacterial culture. Up to ten unstained slides were obtained from the formalin-fixed, paraffin-embedded (FFPE) diagnostic pathology specimen. Slides were shipped to the United States (US) for secondary pathology review.

### Participant treatment and follow-up

Study participants were treated as per institutional protocols and all treatment decisions were made by the treating clinicians. As HL cases were enrolled at the time of referral to Haematology, some participants experienced clinical deterioration and did not receive chemotherapy. First-line treatment for HL in South Africa is ABVD (doxorubicin, bleomycin, vinblastine and dacarbazine). If baseline EF < 50% or concern for compromised respiratory function due to concurrent TB, participants received BCVPP (carmustine, cyclophosphamide, vinblastine, procarbazine, and prednisone). All PWH were treated with concurrent ART. Prophylaxis for PWH includes trimethoprim-sulfamethoxazole 160/800 mg daily if CD4 count is < 200 cells/mm^3^, dose is reduced to three times weekly if significant cytopenias are present. Ciprofloxacin 500 mg twice daily and fluconazole 200 mg daily are added when the absolute neutrophil count < 0.5 × 10^9^/L. Radiation therapy, while available, is used sparingly as the demand within the public sector exceeds the availability. Second-line therapy for relapsed or refractory disease is dose-escalated BEACOPP (bleomycin, etoposide, doxorubicin, cyclophosphamide, vincristine, procarbazine and prednisone). Treatment response is determined by either CT or FDG-PET/CT. Follow-up visits occurred every 2–3 months while on chemotherapy and included a review of clinical notes, laboratory data and chemotherapy administration. For participants non-adherent with Haematology follow-up, telephonic follow-up visits were conducted by the study team. The primary outcome was 1-year overall survival (OS) from time of diagnosis. A secondary outcome was OS from time of chemotherapy initiation. Participants were followed for up to 18 months, until death, loss to follow-up or administrative censoring on Dec 23, 2023. Cause of death was determined through review of medical records including hospital death summaries. Response assessment followed Lugano criteria [16].

### Statistical analysis

Wealth quintiles were calculated as previously described [18, 19]. Data are summarized using descriptive statistics (continuous data) or contingency tables (categorical data) for demographic and baseline characteristics. Either the two sample Wilcoxon test for continuous factors or the Pearson’s chi-squared test for categorical variables was used to compare baseline and other characteristics between groups. Spearman’s correlation was used for the analysis of correlations between continuous variables with non-normal distributions.

OS was calculated from the time of diagnosis, or from the time of chemotherapy. Event time distributions were estimated with the method of Kaplan and Meier [20] and confidence intervals were calculated using the Brookmeyer and Crowley method [21]. Median follow-up is reported using the reverse Kaplan–Meier method. Risk of OS was determined using Cox proportional hazards regression model fitting both univariable and multivariable models. All variables with a *p*-value < 0.1 at the univariable level were considered for inclusion in the multivariable model and the final model was determined using the stepwise selection procedure. Model fit was assessed by the graphical and numerical methods and the final multivariate model was found not to have violated the proportional hazards assumption. All *p*-values reported are two-sided, and the significance level was set at 0.05 for all analyses. Statistical analyses were performed using R version 4.3.1 and SAS Enterprise Guide 7.15 (SAS Institute Inc., Cary, NC, USA).

### Role of the funding source

The funder of the study had no role in the study design, data collection, data analysis, data interpretation, or writing of the report.

## Results

Among the 47 participants with classical HL, 16 participants were HIV-negative and 31 were PWH (Table 1). The majority of participants were recruited from CHBAH (46/47). Wealth quintiles showed a similar distribution regardless of HIV status. The median age was 40 years (Interquartile range (IQR): 30–42) and 24 (51%) participants were male. Among PWH (Table 1), the median CD4 count was 103 (IQR: 40–152 cells/mm^3^) with 19% (6/31) with CD4 < 50 cells/mm^3^ and the majority (n = 25; 81%) had a viral load < 200 copies/mL. Twenty-eight (90%) PWH were on ART at time of HL diagnosis, 1 participant was newly diagnosed with HIV and 2 participants were non-adherent to ART at time of diagnosis and subsequently started on ART. Most PWH on ART (22/28; 79%) had been on ART for > 1 year and the majority of PWH (22/28; 79%) were on tenofovir, lamivudine, dolutegravir (TLD). The median duration of HIV infection was 8.4 years (IQR: 3.7–13.7 years).

**Table 1:** Demographics and Baseline Variables

	Total cohort (N = 47)	HIV (-) (N = 16)	HIV ( +) (N = 31)	*p*-Value
Age, years (IQR)	40.0 (33.5 – 47.5)	39.5 (30.5 – 55.8)	40.0 (34.5 – 45.5)	0.965^1^
Sex (n)				
Male	51% (24)	50% (8)	52% (16)	0.917^2^
Female	49% (23)	50% (8)	48% (15)	
Race (n)				
Black	87% (41)	75% (12)	94% (29)	0.044^2^
White	6% (3)	19% (3)	0% (0)	
Coloured	6% (3)	6% (1)	6% (2)	
Wealth Quintile (n)				
1	21% (7)	20% (2)	22% (5)	0.984^2^
2	15% (5)	20% (2)	13% (3)	
3	21% (7)	20% (2)	22% (5)	
4	24% (8)	20% (2)	26% (6)	
5	18% (6)	20% (2)	17% (4)	
ECOG PS (n)				
0 or 1	45% (21)	50% (8)	42% (13)	0.598^2^
> 2	55% (26)	50% (8)	58% (18)	
B Symptoms (n)				
Present	98% (46)	94% (15)	100% (31)	0.159^2^
Absent	2% (1)	6% (1)	0% (0)	
Stage (n)				
1 or 2	13% (6)	25% (4)	6% (2)	0.071^2^
3 or 4	87% (41)	75% (12)	94% (29)	
Bone Marrow (n)				
Involved	47% (22)	12% (2)	65% (20)	< 0.001^2^
Un-involved	53% (25)	88% (14)	35% (11)	
IPS (n)				
< 4	38% (18)	62% (10)	26% (8)	
≥ 4	62% (29)	38% (6)	74% (23)	0.014^2^
CD4, cells/μL (IQR)	–	–	103.0 (59.0–290.0)	–
HIV Viral Load, copies/mL (n)				
< 200	–	–	81% (25)	
≥ 200			19% (6)	–
ART (n)				
Yes	–	–	90% (28)	–
No			10% (3)	
WBC,10^9^/L (IQR)	4.6 (3.1–11.3)	11.6 (5.2–14.9)	3.8 (2.6–8.7)	0.002^1^
Hemoglobin, g/dL (IQR)	8.9 (7.2–10.9)	11.2 (9.5–13.0)	7.9 (6.4–9.2)	< 0.001^1^
Platelet, 10^9^/L (IQR)	304.0 (96.0–511.5)	428.5 (300.8–516.8)	197.0 (40.5–452.0)	0.057^1^
ALC, 10^9^/L (IQR)	0.9 (0.4–1.5)	1.5 (1.1–2.2)	0.5 (0.3–1.1)	< 0.001^1^
Albumin, g/L (IQR)	31.0 (24.0–35.5)	38.0 (33.8–42.0)	26.0 (23.0–32.0)	< 0.001^1^
TB Treatment (n)				
No	83% (39)	94% (15)	77% (24)	
Yes	17% (8)	6% (1)	23% (7)	0.158^2^
EBER Available (n)				
Yes	60% (28)	56% (9)	61% (19)	–
No	40% (19)	44% (7)	39% (12)	
EBER Evaluable (n)				
Positive	68% (19)	33% (3)	84% (16)	0.007^2^
Negative	32% (9)	67% (6)	16% (3)	

For continuous variables, the median and IQR are shown. N is the number of non–missing values and n are frequencies. Numbers after percents are frequencies. Tests used: ^1^Wilcoxon test; ^2^Pearson’s chi-squared test test. Abbreviations: IQR: Interquartile range; ECOG PS: Eastern Cooperative Oncology Group Performance Status; IPS: International Prognostic Score; ART: Anti-retroviral therapy; WBC: White blood cell; ALC: Absolute lymphocyte count; TB: Tuberculosis

Forty-one participants (87%) had either stage 3 or 4 disease and 46 (98%) presented with B-symptoms (Table 1). Twenty-six participants (55%) had a PS ≥ 2 and 29 (62%) had an IPS of ≥ 4. PWH were more likely to have an IPS ≥ 4 (74% vs 38%: *p* = 0.014). There was no statistical difference between PWH and HIV-negative participants with respect to age, sex, stage, or PS at time of HL diagnosis.

PWH had lower hemoglobin (*p* < 0.001), white blood cell count (*p* = 0.002), absolute lymphocyte count (*p* < 0.001) and albumin (*p* < 0.001). Seven PWH (23%) and one HIV-negative participant (6%) were receiving treatment for active TB disease. The diagnosis of TB was confirmed on sputum GeneXpert (3/8; 37.5%), urine LAM (4/8; 50%) or ZN stain on BMB (1/8; 12.5%). A prior history of TB treatment was noted in 12/31 (38.7%) PWH and none of the HIV-negative participants.

**Fig. 2.**
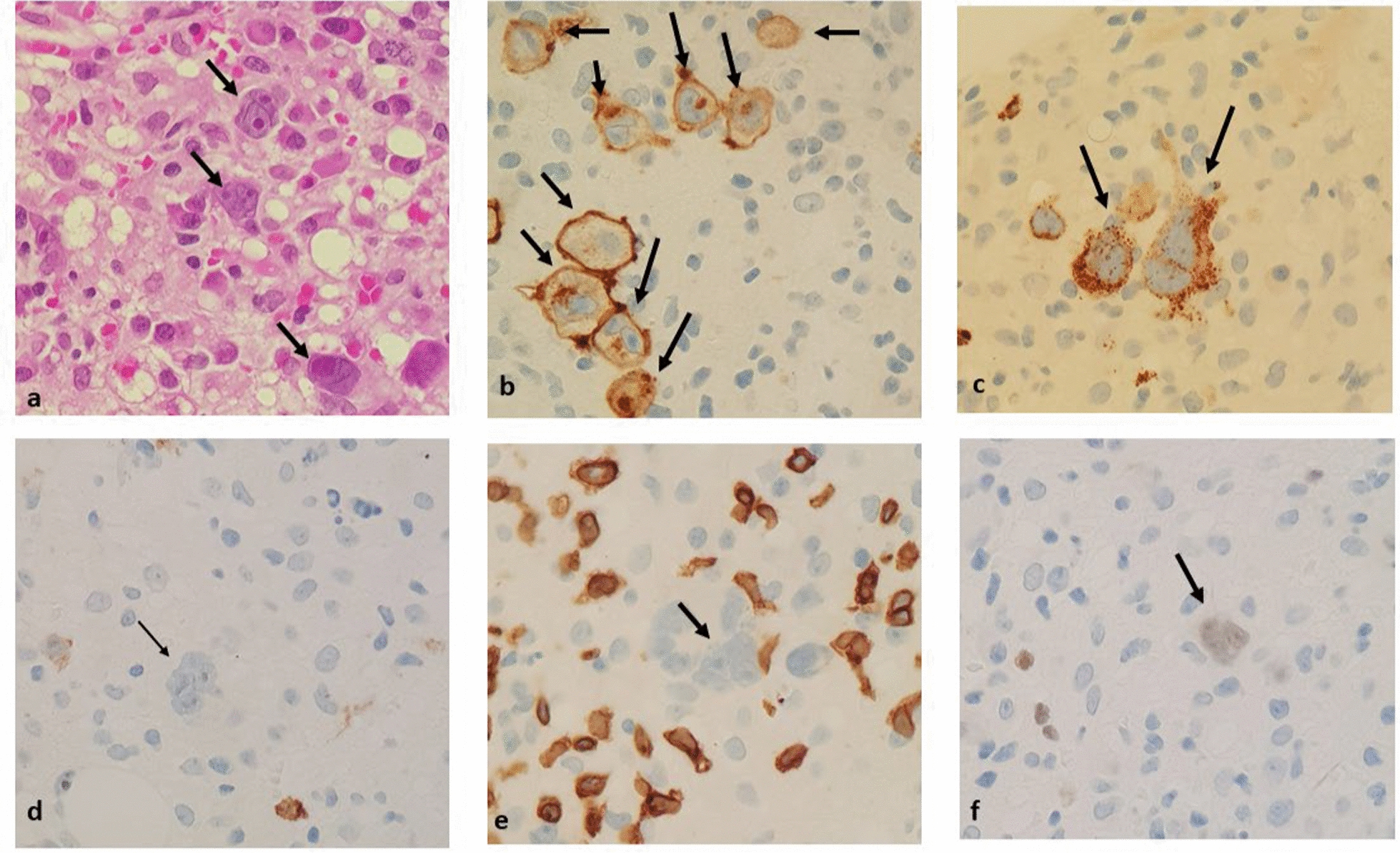
Images from the bone marrow trephine biopsy of one of the patients included in this study. **A**: Demonstrating multiple tumor cells (black arrows), including a classical Reed-Sternberg cell, distributed in a heterocellular inflammatory background. Stained with hematoxylin and eosin, 400× magnification. **B**: Immunohistochemistry for CD30, 400× magnification. Demonstrating positive staining in the tumor cells (black arrows) with a membrane and paranuclear dot-type pattern. **C**: Immunohistochemistry for CD15, 400× magnification. Demonstrating granular cytoplasmic staining in the tumor cells (black arrows). **D**: Immunohistochemistry for CD20, 400× magnification. Demonstrating negative staining in a tumor cell (black arrow). **E**: Immunohistochemistry for CD3, 400× magnification. Demonstrating negative staining in a tumor cell (black arrow). **F**: Immunohistochemistry for PAX5, 400× magnification. Demonstrating weak nuclear staining in a tumor cell (black arrow)

HL histologic subtype was determined for 31 participants with 26 cases of nodular sclerosis (14 PWH; 12 HIV-negative) and 5 cases of mixed cellularity (3 PWH; 2 HIV-negative). In 13 cases, BMB provided the only diagnostic specimen and thus subtype could not be assigned. Sixteen PWH (52%) and 1 HIV-negative participant (6%) had an initial diagnosis confirmed on BMB. Figure 2 shows the representative IHC profile used to diagnosis HL in the BM from 1 participant. Marrow involvement (Table 1) was detected in 22 participants (20 PWH; 2 HIV-negative) and was more common in PWH (65% vs. 13%; *p* = 0.001). The histological profile for all HL cases is included in Supplementary Table 1. Tumor EBV status (Table 1) was evaluable for 28 participants (60%) with 19 EBER- positive HLs (68%). PWH were more likely to have EBV-positive tumors as opposed to HIV-negative participants (84% vs. 33%; p = 0.007).

Marrow involvement (Table 2) was associated with male sex (*p* = 0.028), older age (*p* = 0.045) and HIV status (*p* < 0.001). The majority of PWH with marrow involvement had an HIV viral load < 200 copies/mL (n = 18; 90%), and were noted to have a lower mean CD4 count (*p* < 0.001). White blood cell count (*p* < 0.001), hemoglobin (*p* < 0.001), platelet count (*p* < 0.001), absolute lymphocyte count (*p* < 0.001), and albumin (*p* < 0.001) were all lower among those with marrow involvement.

**Table 2:** Demographics and Baseline Variables by bone marrow involvement

	Bone Marrow Involved (N = 22)	Bone Marrow un-Involved (N = 25)	*p*-Value
Age, year's (IQR)	42.0 (39.0–47.8)	34.0 (32.0–44.0)	0.045^1^
Sex (n)			
Male	68% (15)	36% (9)	0.028^2^
Female	32% (7)	64% (16)	
Race (n)			
Black	91% (20)	84% (21)	0.204^2^
White	0% (0)	12% (3)	
Coloured	9% (2)	4% (1)	
ECOG PS (n)			
0 or 1	32% (7)	56% (14)	0.096^2^
≥ 2	68% (15)	44% (11)	
B Symptoms (n)			
Present	100% (22)	96% (24)	0.343^2^
Absent	0% (0)	4% (1)	
Stage (n)			
1 or 2	0% (0)	24% (2)	0.014^2^
3 or 4	100% (22)	76% (29)	
HIV status (n)			
Positive	91% (20)	44% (11)	< 0.001^2^
Negative	9% (2)	56% (14)	
IPS (n)			
< 4	0% (0)	72% (18)	< 0.001^2^
≥ 4	100% (22)	28% (7)	
CD4, cells/μL (IQR)	74.5 (48.0–110.8)	299.0 (265.5–468.0)	< 0.001^1^
HIV Viral Load, copies/mL (n)			
< 200	90% (18)	64% (7)	0.075^2^
≥ 200	10% (2)	36% (4)	
TB Treatment (n)			
No	73% (16)	92% (23)	0.079^2^
Yes	27% (6)	8% (2)	
WBC, 10^9^/L (IQR)	2.9 (2.0–5.3)	10.3 (4.6–14.4)	< 0.001^1^
Hemoglobin, g/dL (IQR)	7.8 (6.1–8.5)	9.9 (9.3–12.0)	< 0.001^1^
Platelet, 10^9^/L (IQR)	91.0 (29.8–182.8)	495.0 (306.0–587.0)	< 0.001^1^
ALC, 10^9^/L (IQR)	0.5 (0.3–0.6)	1.4 (1.1–2.1)	< 0.001^1^
Albumin, g/L (IQR)	24.5 (22.2–28.8)	35.0 (31.0–41.0)	< 0.001^1^
EBER Evaluable (n)			
Positive	93% (14)	38% (5)	0.002^2^
Negative	7% (1)	62% (8)	

For continuous variables, the median and IQR are shown. N is the number of non–missing values and n are frequencies. Tests used: ^1^Wilcoxon test; ^2^Pearson’s chi-squared test test. Abbreviations: IQR: Interquartile range; ECOG PS: Eastern Cooperative Oncology Group Performance Status; IPS: International Prognostic Score; TB: Tuberculosis; WBC: White blood cell; ALC: Absolute lymphocyte count

Among the 8 participants (Supplementary Table 2) with confirmed TB disease and started on treatment, there was no difference in age, sex, PS, stage, HIV status or CD4 count as compared to those without TB. Those with TB disease were more likely to have an IPS ≥ 4 (100% vs. 54%: *p* = 0.014). They were also noted to have lower hemoglobin (*p* = 0.013) and albumin levels (*p* = 0.04) as compared to those without concurrent TB.

Six participants (5 PWH; 1 HIV-negative) experienced significant clinical deterioration and died prior to receiving chemotherapy. Among participants that received less than 6 cycles of chemotherapy, 10 participants died and 3 participants were non-adherent with Haematology follow-up. The majority of participants received 6 or more cycles of chemotherapy (Table 3; n = 28; 60%). Twenty-seven (14 PWH; 13 HIV-negative) participants were evaluable for end-of-treatment response assessment as 1 participant that received at least 6 cycles of chemotherapy did not undergo imaging to assess response to treatment. Among participants that were evaluable for end-of-treatment response assessment, 17 (63%) experienced a complete response (CR), 6 (22%) had a partial response (PR) and 4 (15%) had progressive disease (PD). There was no difference in treatment response between PWH and HIV-negative participants. Treatment delays among evaluable participants occurred in 8/14 (57%) PWH and 3/13 (23%) HIV-negative participants (*p* = 0.072). Two participants received radiation therapy: 1 participant received radiation to a spinal mass prior to starting chemotherapy and 1 participant received radiation to an inguinal lymph node after chemotherapy.

**Table 3:** Treatment characteristics and response to first-line chemotherapy

	Total cohort	HIV (-)	HIV ( +)	*p*-Value
All participants	N = 47	N = 16	N = 31	
Number of chemo cycles (n)				
No chemo	13% (6)	6% (1)	16% (5)	0.094^1^
< 6 cycles	28% (13)	13% (2)	35% (11)	
≥ 6 cycles	60% (28)	81% (13)	48% (15)	
End of treatment response assessment (n)				
CR	36% (17)	50% (8)	29% (9)	0.209^1^
PR—> 2nd line	13% (6)	19% (3)	10% (3^*^)	
PD—> 2nd line	9% (4)	13% (2)	6% (2)	
Unevaluable	9% (4)	6% (1)	10% (3)	
Death	34% (16)	13% (2)	45% (14)	
*Evaluable for end of treatment response assessment*	N = 27	N = 13	N = 14	
Evaluable end of treatment response assessment^2^ (n)				
CR	63% (17)	62% (8)	64% (9)	0.989^1^
PR—> 2nd line	22% (6)	23% (3)	21% (3^*^)	
PD—> 2nd line	15% (4)	15% (2)	14% (2)	
Treatment delays among evaluable^3^ (n)				
Yes	41% (11)	23% (3)	57% (8)	0.072^1^
No	59% (16)	77% (10)	43% (6)	

N is the number of non–missing values and n are frequencies. ^1^Pearson’s chi-squared test. CR: complete response; PR: partial response; PD: progressive disease. ^2^Evalauble participants included those participants that completed chemotherapy and underwent response assessment by either CT or PET/CT. Participants that died and those that were unevaluable (i.e. did not undergo response assessment by imaging) were not included in this category. ^3^Treatment delays were due to either neutropenia or participant non-adherence with therapy. ^*^One Participant did not proceed to second line chemotherapy after achieving a partial response due to treatment non-adherence

**Fig. 3:**
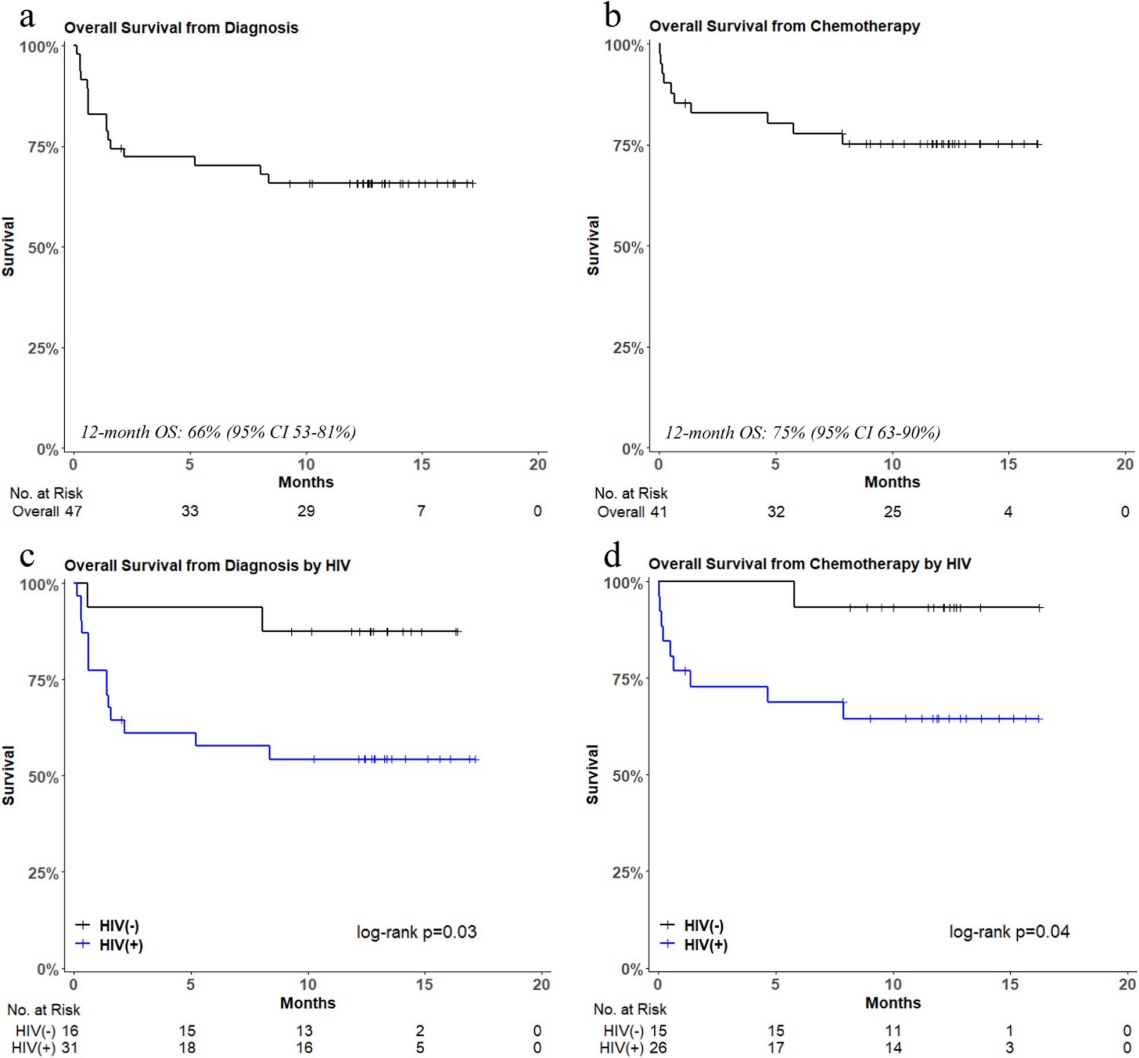
Overall survival from diagnosis and initiation of chemotherapy. *OS* overall survival; *CI* confidence interval; *No. at Risk* number at risk

The 1-year OS was 66% (Fig. 3A). PWH (Fig. 3C) demonstrated poorer 1-year OS, characterized by a marked early mortality (~ 40% mortality within 3 months of diagnosis), as compared to HIV-negative participants (55% vs. 88%; *p* = 0.03). All six participants that did not receive chemotherapy died within 1 month of HL diagnosis. When participants that did not receive chemotherapy (n = 6; 1 HIV-negative, 5 PWH) were excluded from the survival analysis (Fig. 3B & D), 1-year OS improved to 65% for PWH but was still significantly worse as compared to HIV-negative participants (65% vs. 93%; *p* = 0.04).

**Fig. 4:**
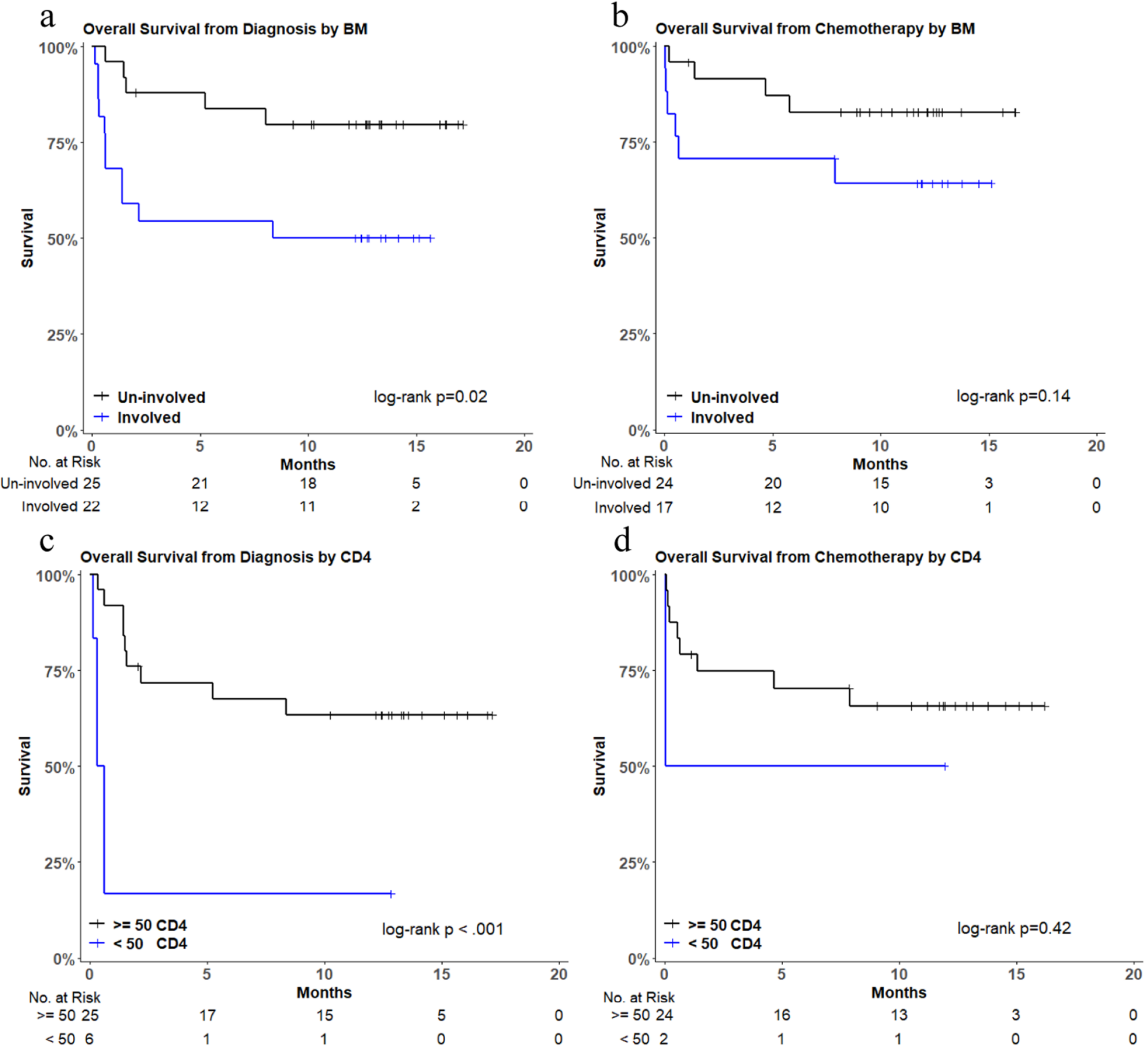
Overall survival by bone marrow involvement and CD4 count from diagnosis and initiation of chemotherapy. *BM* bone marrow; *No. at Risk* number at risk

Participants with marrow involvement were noted to have a worse 1-year OS as compared to those without marrow involvement from time of diagnosis (Fig. 4A). When participants not treated with chemotherapy (n = 6; 5 marrow involved, 1 without marrow involvement) were excluded there was a trend towards worse survival for those with marrow involvement, but no statistical difference (*p* = 0.14; Fig. 4B). Similarly, participants with a CD4 count < 50 cells/mm^3^ (Fig. 4C) had a 1- year OS of 17% compared to 63% in those with a CD4 ≥ 50 cells/mm^3^ (p = 0.0005). Participants that did not receive chemotherapy were overrepresented in the CD4 < 50 cell/mm^3^ (n = 4/5; 80%). When these participants were excluded from the analysis, there was no difference in OS based on CD4 count (Fig. 4D).

In the cox regression analysis (Supplementary Table 3), PWH (HR 4.45 [1.01–19.60]; *p* = 0.05), those with BM involvement (HR 3.33 [1.15–9.61]; *P* = 0.03), and a PS ≥ 2 (HR 3.12 [1.00–9.69]; *p* = 0.05) had an increased risk for death in the univariate analysis. In the multivariate model, none of the parameters were significantly associated with OS.

Clinical characteristics for those patients that died, including HIV status, measures of virologic control, co-morbidities and number of chemotherapy doses received, are listed in Supplementary Table 4. The cause of death for the six participants that did not receive chemotherapy included sepsis (n = 5) and biliary obstruction (n = 1). The remaining ten deaths were attributed to sepsis (n = 5), disease progression (n = 1), acute decompensation after chemo (n = 1), and unknown causes (n = 3). The majority of participant deaths (13/16; 81%) occurred within 3 months from diagnosis in participants receiving < 2 cycles of chemotherapy. The three deaths occurring after 3 months were due to either participant non-adherence to treatment (n = 1) or at home from unknown cause (n = 2), both of these participants experienced infectious complications after chemotherapy.

## Discussion

In this prospective, observational study we report on the clinical characteristics, response to treatment and 1-year OS for classical HL in participants with and without HIV. Participants with marrow involvement had a poor 1-year OS (50%) and disproportionately, PWH with marrow involvement were unable to provide consent to participate due to deteriorating clinical status. While high rates of marrow involvement have previously been reported in PWH [22], diagnosis on BMB is relatively uncommon. In our study, over half of PWH were diagnosed on BMB that was performed during the evaluation of cytopenias. Marrow involvement in the context of HIV appears to convey a particularly poor prognosis in our setting [6]. An interesting observation in PWH and marrow involvement was the combination of a well-controlled HIV viral load and a low CD4 count. In this context we note, HL is associated with lymphopenia regardless of HIV status and several mechanisms have been proposed [23].

The high rates of TB disease in our cohort (22.6% in PWH; 6.3% in HIV-negative) are in keeping with prior reports from South Africa [6, 7]. Participants with TB disease were noted to have lower albumin and hemoglobin levels. The hypoalbuminemia overlaps with levels seen in PWH, while the hemoglobin was roughly 1gm lower. TB has been associated with the development of anemia of chronic inflammation [24]. Given the limited number of cases in our cohort, it is unclear if this represents a clinically significant difference, but it is an important observation to note. Interestingly, we did not see the empiric treatment of TB in our HL cases. This is in contrast to the results of a retrospective review from Cape Town, South Africa where over 50% of PWH received empiric TB treatment [8]. A worse OS in those that were empirically treated for TB was seen in that study, while treatment of confirmed TB disease did not affect survival.

Among participants that were treated with chemotherapy, we report a 65% one-year OS in PWH and 93% in HIV-negative participants. The survival in HIV-negative participants is approaching outcomes seen in advanced stage disease in high-resource settings [25]. Among evaluable participants, the end-of-treatment response was similar for PWH and those without HIV. This suggests that when participants are able to tolerate chemotherapy and remain compliant with follow-up, good clinical outcomes can be achieved regardless of HIV status. Unfortunately, PWH were overrepresented in those receiving < 6 cycles of chemotherapy due to early mortality or participant non-adherence. What remains unclear is whether the advanced presentations seen at time of diagnosis are a manifestation of delayed diagnosis in an overburdened health system or whether a more aggressive biology is occurring in a subset of PWH. Interestingly, data from the US suggests a worse prognosis for PWH and HL with undetermined histology [26]. Over 1/3 of our participants had undetermined histology due to the diagnosis of HL on BMB. Furthermore, an increased risk of death attributable to HIV has been reported in large observational studies of HL in Italy [2] and the US [11], and continued disparity in receipt of chemotherapy for HIV-associated HL has been noted [12]. Altogether, real-world outcomes continue to lag behind those from clinical trials for PWH.

In the backdrop of a hyper-TB epidemic with one of the highest rates of HIV worldwide, in South Africa there appears to be a subset of PWH presenting with an overly aggressive clinical course that is occurring in a higher number, although with similar outcomes, to real-world data from high-resource settings. The well documented association between EBV and HL in PWH [27, 28], confirmed in our study, and the evolving research on the clinical impact of EBV DNA methylation patterns offers an exciting substrate for future research [29].

When comparing our results to other prospective data from the continent, (Botswana [13] and Malawi [14]) similar survival between PWH and HIV-negative individuals has been reported. Similar to the Malawi study, we also note more treatment delays during chemotherapy among PWH that did not translate into poorer treatment outcomes. However, there are several key differences to note. The Botswana study excluded participants unable to provide consent and BMB data was not available. Given that the majority of PWH were diagnosed on BMB in our setting, lack of access to BMB could limit HL diagnosis in Botswana, and given that marrow involvement was associated with a particularly poor survival, this difference could affect a comparison of survival outcomes between the two studies. No mention of diagnosis on BMB was noted in the Malawi study. We also included a family consent for participants and note a disproportionate number of PWH not enrolled in our cohort due to early mortality or urgent need for chemotherapy. Lastly, advanced stage and poor PS were universal in our setting regardless of HIV status, while participants with early stage disease were seen more frequently in both Botswana and Malawi. This could suggest that diagnostic delays are more common in the South African public healthcare sector, where the majority of our participants were recruited. Our findings also confirm the poor treatment outcomes from previous retrospective studies from South Africa [8] and Tanzania [30].

Our study has several limitations. The small sample size resulted in a small number of events for several variables of interest and this can affect our power to detect differences among groups. Our sample size, is however, in keeping with the other prospective data from sub-Saharan Africa [13, 14]. Another important consideration when comparing our results to other studies is the overlap with participant enrollment and the early Covid pandemic. Three participant deaths occurred in the context of a Covid infection, two PWH developed gram-negative sepsis and one HIV-negative participant had been hospitalized for pneumonia and a recurrent pleural effusion, and died at home of unknown cause.

A major strength of our study is the reporting of real-world treatment outcomes for HL in South Africa. These results are in contrast to clinical trial [10] and observational data [9] from high-resource settings showing the successful treatment of advanced stage HIV-associated HL with ABVD chemotherapy. Unfortunately, these studies would have excluded a large number of the participants referred to our study team due to stringent eligibility criteria for clinical trials and requirement for consent for observational studies. We do, however, note an 85% overall response rate (63% CR; 22% PR) for participants that were eligible for response assessment. In high-resource settings, the current focus of HL treatment is to move the needle by improving durable CR rates for clinical trial eligible participants with the incorporation of novel agents such as Brentuximab Vedotin [31] and immune checkpoint inhibitors [32, 33]. The more pressing question for PWH with HL worldwide, is how to effectively manage participants that are either unable to tolerate ABVD or are ineligible for clinical trials.

## Conclusions

Advanced stage disease presentations were universal in our study, regardless of HIV status, and highlight the need to better understand the drivers of diagnostic delay in this setting. In Johannesburg, a large percentage of PWH presenting with lymphoma do not survive long enough to have their lymphoma diagnosis confirmed [6, 34] and we can confirm that many others are too ill to receive chemotherapy at time of diagnosis. The current diagnostic paradigm in low-resource settings appears inadequate to accommodate the increasing burden of hematologic malignancies [35]. Strategies to improve the triage of high-risk individuals through the healthcare system to facilitate lymphoma diagnosis are needed. At the patient level, a better understanding of the social determinants of health that contribute to participant non-adherence, another common barrier to care, is warranted. Initiatives focused on biomarker development to improve the diagnosis of HL and to better identify those at high-risk for early mortality could prove extremely beneficial worldwide, and could help shed light on the aggressive biology that was observed in our cohort.

## Supplementary Information

Additional file 1.

## Data Availability

The deidentified data set, data dictionary, and study protocol are available upon request sent to the corresponding author (svogt2@jhmi.edu).

## Abbreviations

HL: Hodgkin lymphoma
ART: Antiretroviral therapy
TB: Tuberculosis
PWH: People with HIV
CHBAH: Chris Hani Baragwanath Academic Hospital
ECOG: Eastern cooperative oncology group
PS: Performance status
IHC: Immunohistochemistry
BMB: Bone marrow biopsy
EF: Ejection fraction
IPS: International prognostic score
ZN: Ziehl–Neelsen
LAM: Lipoarabinomannan
FFPE: Formalin-fixed, paraffin-embedded
US: United States
ABVD: Doxorubicin, bleomycin, vinblastine and dacarbazine
BCVPP: Carmustine, cyclophosphamide, vinblastine, procarbazine, and prednisone
BEACOPP: Bleomycin, etoposide, doxorubicin, cyclophosphamide, vincristine, procarbazine and prednisone
OS: Overall survival
IQR: Interquartile range
TLD: Tenofovir, lamivudine, dolutegravir
CR: Complete response
PR: Partial response
PD: Progressive disease
