# Supplementary material for: Real-world treatment outcomes for Hodgkin lymphoma in South Africa: a prospective observational study
Source: Infect Agent Cancer. 2024 Sep 27;19:46. doi: 10.1186/s13027-024-00612-4 (PMC11428538; doi:10.1186/s13027-024-00612-4)
Supplement: Supplementary file 1 — Additional file 1. [file 13027_2024_612_MOESM1_ESM.docx]

**Supplementary Data**

**Supplementary Table 1. Pathologic evaluation and immunophenotype**.

| **ID** | **CD3** | **CD20** | **CD30** | **CD15** | **PAX5** | **MUM1** | **CD45** | **ALK1** | **EBER** | **HIV status** | **BM** | **LN** | **Dx Tissue** |
| --- | --- | --- | --- | --- | --- | --- | --- | --- | --- | --- | --- | --- | --- |
| 1 | - | - | + | - | + (weak) | . | . | . | . | - | - | + | LN |
| 2 | - | - | + | - | + (weak) | + | . | . | - | - | - | + | LN |
| 3 | - | - | + | - | . | + | . | . | - | - | - | + | LN |
| 4 | - | - | + | + (focal) | + (weak) | - | . | . | + | - | - | + | LN |
| 5 | - | - | + | + | + (weak) | . | - | . | + | - | - | + | LN |
| 6 | - | - | + | + | - | . | . | . | - | - | - | + | LN |
| 7 | . | + (focal) | + | + | + (weak) | . | - | . | . | - | - | + | LN |
| 8 | - | - | + | + | + (weak) | . | . | . | . | - | - | + | LN |
| 9 | - | - | + | + | + (weak) | . | . | . | - | - | - | + | LN |
| 10 | - | - | + | + | + (weak) | . | - | . | + | - | + | + | LN |
| 11 | . | + (weak) | + | + | + (weak) | . | . | . | . | - | - | + | LN |
| 12 | . | . | + | + | + | . | . | . | . | - | + | - | BM |
| 13 | - | - | + | + | + (weak) | . | . | . | . | - | - | + | LN |
| 14 | - | - | + | + | + (weak) | + | . | . | - | - | - | + | LN |
| 15 | - | - | + | + | . | + | . | - | . | - | - | + | LN |
| 16 | - | - | + | + | + (weak) | . | . | . | - | - | - | + | LN |
| 17 | - | + | + | + (focal) | + | + | + | . | + | + | - | + | LN |
| 18 | - | - | + | - | + (weak) | + | - | - | - | + | - | + | LN |
| 19 | - | - | + | + | + (weak) | . | . | . | + | + | - | + | LN |
| 20 | - | . | + | + | + (weak) | . | . | . | . | + | + | + | BM |
| 21 | - | - | + | - | + | + | - | . | + | + | + | + | BM |
| 22 | . | - | + | + (weak) | + (weak) | + | . | . | . | + | + | - | BM |
| 23 | - | + (variable) | + | + | + (weak) | . | - | . | - | + | - | + | LN |
| 24 | - | - | + | + | . | . | . | . | + | + | + | - | BM |
| 25 | - | + (focal) | + | + (weak) | + (weak) | . | . | . | . | + | + | - | BM |
| 26 | - | - | + | + (focal) | - | + (weak) | - | - | . | + | + | + | BM |
| 27 | - | - | + | + | + (weak) | . | . | . | + | + | - | + | LN |
| 28 | - | - | + | + | . | . | . | . | . | + | - | + | LN |
| 29 | - | + (weak) | + | + (weak) | + (weak) | . | . | . | + | + | + | - | BM |
| 30 | - | - | + | + | + (weak) | . | . | . | + | + | + | - | BM |
| 31 | - | - | + | + | + (weak) | . | . | . | + | + | + | - | BM |
| 32 | . | + (focal) | + | + | + (weak) | . | - | . | . | + | + | + | LN |
| 33 | - | - | + | + (focal) | + (weak) | . | . | . | + | + | + | - | BM |
| 34 | - | + (weak) | + | + | + (weak) | . | - | . | . | + | - | + | LN |
| 35 | - | - | + | + (focal) | + (weak) | . | - | . | . | + | + | - | BM |
| 36 | - | + (focal) | + | + | + (weak) | . | . | . | + | + | + | - | BM |
| 37 | - | - | + | + | + (weak) | + (weak) | . | . | . | + | - | + | LN |
| 38 | - | + (weak) | + | + (variable) | + (weak) | . | . | . | + | + | + | + | LN |
| 39 | - | + (weak) | + (variable) | + | + (weak) | . | . | . | + | + | + | + | BM |
| 40 | - | - | + | - | - | . | . | - | + | + | + | - | BM |
| 41 | - | + (focal) | + | + (variable) | + (weak) | . | . | . | . | + | + | - | BM |
| 42 | - | - | + | + | + | . | - | . | . | + | - | + | LN |
| 43 | - | - | + | + | - | + | . | - | + | + | + | + | BM |
| 44 | . | - | + | + | + (weak) | . | . | . | . | + | - | + | LN |
| 45 | . | - | + | + | + (weak) | . | . | . | . | + | + | + | LN |
| 46 | - | - | + | + | + | . | . | . | . | + | - | + | LN |
| 47 | - | - | + | + | . | + | . | - | + | + | + | + | LN |

Abbreviations: BM: bone marrow; LN: lymph node; Dx: diagnostic; (-): negative or not involved; (+): positive or involved; (.): not performed. *A diagnosis of grey zone lymphoma was considered for HL010, but was not favored due to the histology that was typical for nodular sclerosis subtype of classical Hodgkin lymphoma, EBER positivity and the extent of nodal involvement.^1^

**Supplementary Table 2. Demographics and Baseline Variables by Tuberculosis disease status.**

|  | **TB Disease**  **(N = 8)** | **No TB**  **(N = 39)** | **p-Value** |
| --- | --- | --- | --- |
| Age, years (IQR) | 41.0 (38.8 – 44.8) | 40.0 (33.0 – 48.0) | 0.72^1^ |
| Sex (n)  Male  Female | 38% (3)  62% (5) | 54% (21)  46% (18) | 0.4^2^ |
| Race (n)  Black  White  Coloured | 75% (6)  13% (1)  13% (1) | 90% (35)  5% (2)  5% (2) | 0.52^2^ |
| ECOG PS (n)  0 or 1  > 2 | 25% (2)  75% (6) | 49% (19)  51% (20) | 0.22^2^ |
| B Symptoms (n)  Present  Absent | 100% (8)  0% (0) | 97% (38)  3% (1) | 0.65^2^ |
| Stage (n)  1 or 2  3 or 4 | 0% (0)  100% (8) | 15% (6)  85% (33) | 0.24^2^ |
| HIV status (n)  Positive  Negative | 88% (7)  12% (1) | 62% (24)  38% (15) | 0.16^2^ |
| IPS (n)  < 4  > 4 | 0% (0)  100% (8) | 46% (18)  54% (21) | 0.01^2^ |
| CD4, cells/μL (IQR) | 103.0 (72.0 – 113.5) | 127.0 (51.0 – 303.8) | 0.96^1^ |
| HIV Viral Load, copies/mL (n)  < 200  > 200 | 86% (6)  14% (1) | 79% (19)  21% (5) | 0.7^2^ |
| WBC, 10^9^/L (IQR) | 3.4 (2.7 – 10.0) | 5.4 (3.4 – 11.3) | 0.62^1^ |
| Hemoglobin, g/dL (IQR) | 6.8 (5.4 – 8.2) | 9.3 (7.4 – 11.3) | 0.01^1^ |
| Platelet, 10^9^/L (IQR) | 106.0 (40.2 – 199.0) | 324.0 (128.5 – 517.5) | 0.08^1^ |
| ALC, 10^9^/L (IQR) | 0.5 (0.4 – 0.8) | 1.0 (0.4 – 1.5) | 0.44^1^ |
| Albumin, g/L (IQR) | 25.0 (22.2 – 27.5) | 32.0 (24.5 – 38.5) | 0.04^1^ |

For continuous variables, the median and IQR are shown. N is the number of non–missing values and n are frequencies. Tests used: ^1^Wilcoxon test; ^2^Pearson’s chi-squared test. Abbreviations: IQR: Interquartile range; TB: Tuberculosis; ECOG PS: Eastern Cooperative Oncology Group Performance Status; IPS: International Prognostic Score; WBC: White blood cell; ALC: Absolute lymphocyte count

**Supplementary Table 3. Univariate and multivariate cox proportional hazard estimated for overall survival.**

| **Variable** | **Value** | **Univariate**  **HR (95% CI)** | **p-Value** | **Multivariate**  **HR (95% CI)** | **p-Value** |
| --- | --- | --- | --- | --- | --- |
| HIV status | Positive | 4.45 (1.01-19.63) | 0.05 | 3.17 (0.63-15.91) | 0.16 |
| Bone Marrow Involvement | Involved | 3.33 (1.15-9.61) | 0.03 | 1.88 (0.59-5.97) | 0.29 |
| ECOG PS | >2 | 3.12 (1.00-9.69) | 0.05 | 2.89 (0.92-9.05) | 0.07 |
| Sex | Male | 0.77 (0.29-2.07) | 0.60 | - |  |
| Age | >45 years | 1.01 (0.98-1.06) | 0.47 | - |  |
| Stage | III-IV | 2.42 (0.32-18.31) | 0.39 | - |  |
| IPS | >4 | 5.56 (1.26-24.51) | 0.02 | - |  |
| Albumin | - | 0.90 (0.84-0.97) | 0.004 | - |  |
| ALC | - | 0.35 (0.13-0.91) | 0.03 | - |  |
| Hemoglobin | - | 0.93 (0.79-1.09) | 0.35 | - |  |
| WBC | - | 1.00 (0.96-1.05) | 0.95 | - |  |

HIV status (positive vs negative), bone marrow involvement (involved vs uninvolved), and ECOG PS (>2 vs <2) were all included in the multivariate analysis. Variables with a value listed were dichotomous while laboratory values for albumin, ALC, hemoglobin and WBC were continuous measures. Abbreviations: HR: hazard ratio; CI: confidence interval; ECOG PS: Eastern Cooperative Oncology Group Performance Status; IPS: International Prognostic Score; ALC: Absolute lymphocyte count

**Supplementary Table 4. Cause of death.**

| **HIV** | **CD4** | **Viral load** | **Chemo** | **Co-morbidity** | **Cause of death** | **OS from diagnosis** |
| --- | --- | --- | --- | --- | --- | --- |
| Not treated with chemotherapy | | | | | | |
| Neg |  |  | None | Prior Mycosis Fungoides, severe neutropenia with ANC 0 on neupogen | In hospital  Neutropenic sepsis | 18 days |
| Pos | 13 | <50 | None | JC virus and abdominal imaging concerning for TB (urine Lam neg x 2), Covid + on oxygen, sputum culture with Klebsiella (CRE) | In hospital  Sepsis 2/2 CRE | 9 days |
| Pos | 48 | 55 | None | Myelopathy with paraplegia, course complicated by urinary retention and hypernatremia | In hospital  Sepsis | 9 days |
| Pos | 48 | <50 | None | Obstructive jaundice | In hospital  Biliary obstruction | 19 days |
| Pos | 12 | 77 | None | Prior history of TB, severe liver disease, pancytopenia, blood culture growing E.coli (CRE) | In hospital  Sepsis 2/2 CRE | 19 days |
| Pos | 52 | <50 | None | Liver and kidney injury, coagulopathy, nosocomial sepsis on broad spectrum antibiotics, pleural effusion and seizures | In hospital  Sepsis | 45 days |
| Being treated for concurrent TB | | | | | | |
| Pos | 66 | <50 | ABVD x 2 doses  (Oct; Jan) | Disseminated TB based on urine Lam, non-adherent to chemo/ART/TB therapy, admitted with jaundice and severe anemia | In hospital  Progressive disease | 255 days |
| Pos | 72 | > 1 million | ABVD x 1 dose | Pulmonary TB on TB treatment complicated by drug induced liver injury, Cdiff + | In hospital  Sepsis | 43 days |
| Pos | 103 | <50 | ABVD x 1 dose | Disseminated TB based on urine Lam, Cdiff +, Covid +, nosocomial sepsis with blood culture growing Klebsiella (CRE) | In hospital  Sepsis 2/2 CRE | 66 days |
| Pos | 499 | <50 | ABVD x 3 doses | Pulmonary TB on TB treatment, admitted with recurrent pleural effusion | In hospital  Unknown cause | 48 days |
| Other deaths | | | | | | |
| Neg |  |  | ABVD x 2 cycles | HTN, Asthma, prolonged hospital admission for Covid pneumonia after receiving cycle 1A, admitted with pneumonia and large pleural effusion 1 month prior to death | At home  Unknown cause | 245 days |
| Pos | 432 | <50 | ABVD x 1 dose | Prior history of TB, pancytopenia (ANC 0, Hgb 7.3, Plts 1) | In hospital  Neutropenic sepsis | 19 days |
| Pos | 508 | 191,000 | ABVD x 2 cycles | Prior history of TB, cycle 2A delayed 2 months due to grade 3 neutropenia, admitted after cycle 2A for peri-orbital cellulitis complicated by grade 3 neutropenia 1 month prior to death | At home  Unknown cause | 167 days |
| Pos | 34 | 104 | ABVD x 1 dose | On INH prophylaxis, pancytopenia, Cdiff +, encephalopathic | In hospital  Sepsis | 4 days |
| Pos | 318 | <50 | ABVD x 1 dose | Acute decompensation after chemotherapy requiring emergent hemodialysis | In hospital  Acute decompensation | 10 days |
| Pos | 93 | 244 | ABVD x 1 dose | Admitted with liver failure, DIC, seizures, pancytopenia | In hospital  Sepsis | 43 days |

Abbreviations: OS: overall survival; Neg: HIV-negative; Pos: HIV-positive; ANC: absolute neutrophil count; JC virus: Human polyomavirus 2; TB: tuberculosis; urine LAM: urine lipoarabinomannan; Covid+: testing positive for Covid 19; CRE: carbapenem-resistant Enterobacteriaceae; ABVD: doxorubicin, bleomycin, vinblastine and dacarbazine; Oct: October; Jan: January; ART: antiretroviral therapy; Cdiff+: Clostridium difficile infection; HTN: hypertension; Hgb: hemoglobin; Plt: platelets; INH: isoniazid; DIC: diffuse intravascular coagulation.

**References**

1. Pilichowska M, Pittaluga S, Ferry JA, et al. Clinicopathologic consensus study of gray zone lymphoma with features intermediate between DLBCL and classical HL. *Blood Adv* 2017; **1**(26): 2600-9.
